# Oral Rehabilitation in Patient With Hereditary Sensory and Autonomic Neuropathy (HSAN) Type V: Clinical Report

**DOI:** 10.1155/crid/6868923

**Published:** 2025-11-07

**Authors:** Sana Lala, Ammar Almustafa

**Affiliations:** Department of Removable Prosthodontic, Faculty of Dental Medicine, Damascus University, Damascus, Syria

## Abstract

Hereditary sensory and autonomic neuropathies (HSANs) are rare inheritable syndromes of unknown etiology. They typically appear in early childhood and are categorized into six different types based on their symptoms. HSAN-V is characterized by loss of pain and thermal perception, Charcot joints, painless fractures, scoliosis, oral lesions, and absent corneal reflexes. The HSAN-V phenotype has a propensity to progress to autoamputation, varying degrees of hypohidrosis, and moderate hyperactivity and perception delay. In this case report, we focused on oral findings, prosthodontic treatments, and oral rehabilitation of a 9-year-old girl with HSAN type V.

## 1. Introduction

Hereditary sensory and autonomic neuropathies (HSANs) are rare heritable syndromes characterized by innate insensitivity to pain and temperature changes and are classified under autonomic nerve disorders [1].

Individual differences in pain perception occur due to complex genetic variability [2]. The absence of pain sensation from birth or throughout life occurs due to specific nucleotide variants, in contrast to environmental and polygenic correlations [2]. Disorders of neurodegeneration of peripheral nerves and altered electrical activity of nociceptors or pain-sensing neurons usually develop in these monogenic and rare diseases [2]. Hereditary sensory neuropathy (HSN), congenital insensitivity to pain (CIP), and HSAN (if autonomic nerves are involved) are a heterogeneous group of genetic pain-loss disorders [2]. The result of losing pain sensation (touch, itch, and temperature) includes fractures causing amputation or mutilation, severely impaired wound healing, and recurrent injuries [2]. Many autonomic dysfunctions can affect patients, such as unstable blood pressure, anhidrosis, sexual dysfunction, and gastrointestinal issues. In some subtypes of CIP/HSAN, patients may complain of ataxia, intellectual disability, muscle weakness, or other symptoms [2]. To date, more than 20 genes with pathogenic variants are known to cause the pain-loss syndromes [2].

Many cellular processes may be affected through changes in neurotrophin signaling [3] and cytoskeletal architecture [4].

These conditions are classified into six types depending on the case's natural history, the population of neurons or axons affected, and the mode of inheritance [1]. Type I is a moderately mild condition, manifested in the second to fourth decade, substantially affecting the lower limbs [5]. Type II appears with loss of perspiration despite normal temperature and blood pressure [5]. Type III has a multisystem presentation including postural hypotension, ataxia, kyphoscoliosis, oropharyngeal dyscoordination, and abnormal gastroesophageal motility resulting in feeding difficulties and intermittent aspiration pneumonia [5]. Type IV manifests in childhood and is associated with pain insensitivity, complete anhidrosis, intellectual disability, and self-mutilation [5]. Type V presents similarly to Type IV but is milder and without intellectual disability [5].

HSAN type V is defined by a lack of pain perception, mental status, and partial anhidrosis, but with preservation of all sensory modalities, including normal touch perception, proprioception, itch, warm and cold temperature discrimination, and vibration [6]. It is caused by a mutation in the neurotrophic tyrosine kinase receptor, type 1 (NTRK1) gene located on chromosome 1 (1q21-q22), coding for nerve growth factor beta (NGFB) [6, 7]. The loss of pain sensation interferes with body protective mechanisms, sleep, mobility, nutrition, cognition, emotional well-being, creativity, and self-actualization [7]. Self-mutilation is a nearly invariable character of this disease, most frequently affecting the teeth, lips, tongue, ears, eyes, nose, and fingers [7]. Bone fractures are also asymptomatic, and affected individuals are unaware of these fractures until a limp develops [7].

Limited diagnosis and knowledge of rare CIP/HSAN disorders often lead to misdiagnosis or delays in the diagnostic process. Additionally, the literature often includes only a single case description, with few studies reporting on larger patient samples [8, 9].

This report outlines a case of HSAN type V, highlighting the oral rehabilitation that improved the chewing function, restored aesthetic appearance, and rebuilt the vertical dimension for the lower third of the face.

## 2. Clinical Report

**Figure 1:**
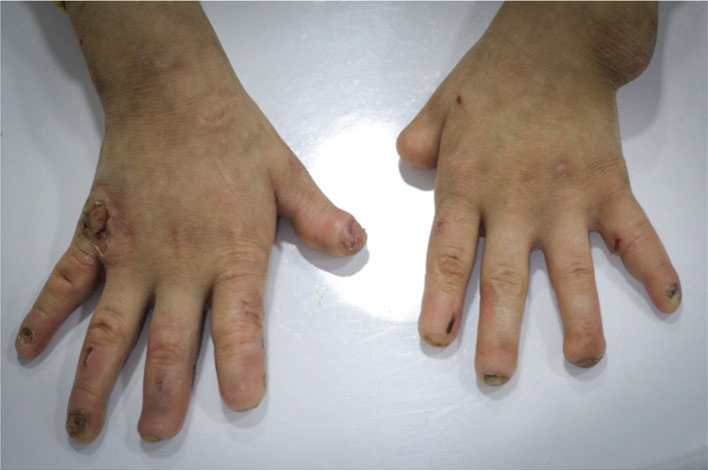
Short and blunt hand fingers.

A 9-year-old girl was referred to the Department of Removable Prosthodontics, Faculty of Dentistry, by her pediatrician with a history of teeth loss and a chief complaint of eating difficulties. She was the seventh child in her family, born without complications, and there was no family history of a similar condition. The patient's medical history revealed that she was clinically diagnosed with HSAN type V at the age of 2 years by her pediatrician based on nonhealing ulcers, inflammatory indices, and angiography CT with MSCT injection, which showed noncongenital inflammatory angiostenosis. Clinical features included nonhealing ulcers, especially in pressure areas (malleolus), autoresorption of terminal phalanges in hands, hypodontia, and maxillary atelognathia. The ulcers became more severe in the lower limbs, leading to inflammation, gangrene, necrosis, and multiple amputations both above and below the knee. She was treated with immune-inhibitor (cyclosporine A), cortisone (Predalone), aspirin 81, warfarin, nifidipine, nitraterm, nitroglecyrine cream, calcium and iron supplements, topical (tacrolimus + ozocure cream), zinc dressing, and multiple sessions of hyperbaric oxygen therapy. General examination revealed normal reactions to touch and pressure, but no response to thermal stimuli or pain. Her fingers were short and blunt, with visible tissue loss (Figure 1).

**Figure 2:**
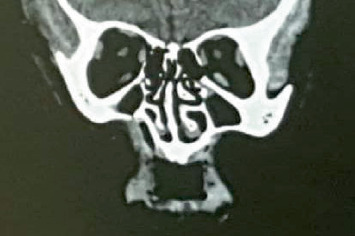
CT scan for the head shows the small hole in the floor of the maxillary sinus in the right side.

**Figure 3:**
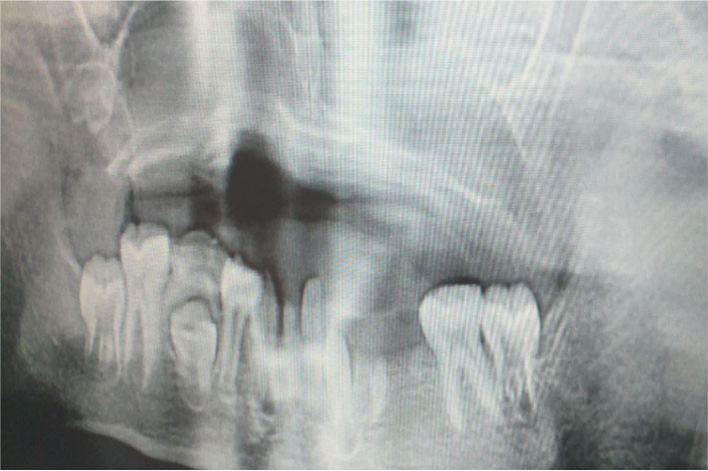
Panoramic picture.

**Figure 4:**
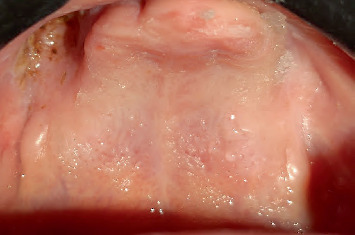
Intraoral view for maxilla.

**Figure 5:**
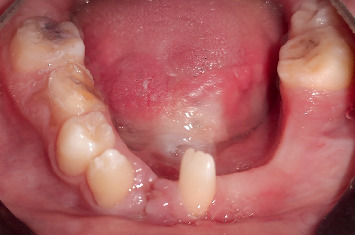
Intraoral view for mandibular.

Intraoral examination showed a deep cavity in the right upper buccal sulcus opening into the maxillary sinus, as confirmed by CT scan (Figure 2). All upper teeth, lower lateral incisors, left canine, and left first and second premolars were missing. According to her family, maxillary teeth were lost spontaneously. The lower left central incisor was impacted, and the lower right second premolar had not yet erupted, as seen on the panoramic radiograph (Figure 3). Severe bone loss was observed in maxillary bone (Figure 4). There was limited mouth opening and tongue ulcers caused by dental irritation (Figure 5).

**Figure 6:**
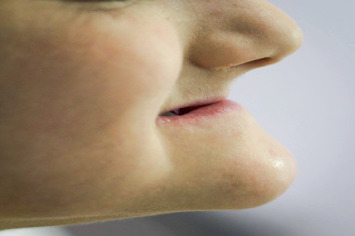
Extra oral view shows the loss in the vertical dimension in the lower third of the face.

**Figure 7:**
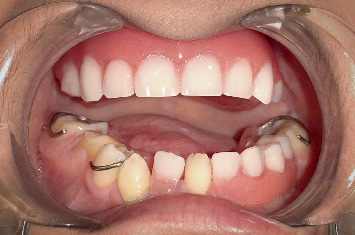
Complete upper and partial lower removable dental prosthesis.

**Figure 8:**
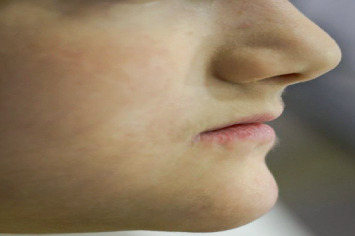
Extraoral view after rebuilding the vertical dimension for the lower third of the face and establish lip support.

Extraoral examination showed loss of vertical dimension in the lower third of the face (Figure 6). The treatment plan focused on oral rehabilitation and restoration of oral function. A complete upper and partial lower removable dental prosthesis was fabricated to close the maxillary sinus opening, improve chewing function (Figure 7), provide lip support, restore aesthetics, and rebuild the vertical dimension for the lower third of the face (Figure 8).

The clinical steps for fabricating the removable prosthesis were carried out in the following sequence: An initial impression was taken with alginate after adjusting the tray. Custom trays were then fabricated using self-cured acrylic resin, and the final impressions were obtained with impression compound for the upper jaw and with alginate for the lower jaw. Base plates with wax rims were constructed, and the maxillomandibular relationship was recorded after adjusting the wax rims to match the clinical condition. In the subsequent appointment, a trial of the artificial teeth was performed to verify lip support, vertical dimension, proper occlusion, and esthetics. Following this, the prosthesis was processed, and the final removable prostheses were delivered to the child.

The child required approximately 1 month to achieve full acceptance of the prostheses. She wears it most of the day and removes it completely during nighttime sleep. Follow-up appointments were scheduled weekly in the first month, then every 15 days during the second month, and subsequently once per month.

After 6 months, the prosthesis was modified to accommodate the minor jaw growth. The parents were informed about the necessity of regular follow-up visits for further adjustments according to the child's growth, and the possibility of fabricating a new prosthesis after a year or more, depending on the rate of jaw development.

The choice of a removable complete denture for the maxilla and a removable partial denture for the mandible was guided by several clinical considerations. The patient presented with extensive alveolar bone loss, complete edentulism in the maxilla, and partial edentulism in the mandible. Given the age of the patient, ongoing growth, and the underlying sensory neuropathy (HSAN Type V), fixed prosthetic options or implant placement were contraindicated. Moreover, removable prostheses allow for easier hygiene maintenance and adjustments, which is critical in patients with compromised pain perception and self-injurious behavior. This approach aligns with recommendations in the literature for prosthodontic management of pediatric patients with neurogenic disorders and severe oral trauma.

Informed consent was obtained from the patient's legal guardian for the dental treatment and the publication of this case report. The guardian explicitly requested that the patient's name and full facial identity not be disclosed, a condition we respected throughout the manuscript.

While institutional review board (IRB) approval is typically not required for single case reports in our institution, this has been acknowledged in the text.

In this case report, a nerve biopsy or genetic test could not be performed, as the patient's family declined to include such data in the manuscript. Therefore, the diagnosis was based on clinical features, biochemical evaluations, and the extent of both sensory and autonomic dysfunction, which has been acknowledged as a limitation of the study.

## 3. Discussion

Children with congenital disorders and syndromes present major challenges in the dental clinic [10]. Most congenital disorders are evident early in life, particularly in premature infants, and require high attention right from birth. HSAN type V is a congenital disorder that can be diagnosed after birth due to pain insensitivity, absence of intellectual disability [11], moderate loss of thin myelinated nerve fibers, and severe reduction of unmyelinated nerve fibers [12].

Rare syndromes like Cornelia de Lange syndrome and Lesch–Nyhan syndrome also present self-mutilation and pain insensitivity. HSAN type V can be distinguished from these by the absence of peripheral nerve thickening and dysmorphic features, electrophysiological studies, partial anhidrosis, normal serum uric acid levels, and histopathological findings showing nerve fiber loss [13].

## 4. Summary

A 9-year-old girl with HSAN received upper complete and lower partial removable prosthesis to restore the chewing functions, esthetic appearance, vertical facial dimension, and lip support, as well as to close the maxillary sinus opening. This treatment helped restore the child's self-confidence. Her parents were well-educated about the importance of regular follow-ups and maintaining good oral hygiene for the girl.

## Data Availability

The data that support the findings of this study are available on request from the corresponding author. The data are not publicly available due to privacy or ethical restrictions.
